# Integrative Approach Reveals Composition of Endoparasitoid Wasp Venoms

**DOI:** 10.1371/journal.pone.0064125

**Published:** 2013-05-23

**Authors:** Jeremy Goecks, Nathan T. Mortimer, James A. Mobley, Gregory J. Bowersock, James Taylor, Todd A. Schlenke

**Affiliations:** 1 Department of Biology, Emory University, Atlanta, Georgia, United States of America; 2 Department of Mathematics and Computer Science, Emory University, Atlanta, Georgia, United States of America; 3 Department of Surgery, University of Alabama-Birmingham, Birmingham, Alabama, United States of America; 4 Comprehensive Cancer Center, University of Alabama-Birmingham, Birmingham, Alabama, United States of America; North Carolina State University, United States of America

## Abstract

The fruit fly *Drosophila melanogaster* and its endoparasitoid wasps are a developing model system for interactions between host immune responses and parasite virulence mechanisms. In this system, wasps use diverse venom cocktails to suppress the conserved fly cellular encapsulation response. Although numerous genetic tools allow detailed characterization of fly immune genes, lack of wasp genomic information has hindered characterization of the parasite side of the interaction. Here, we use high-throughput nucleic acid and amino acid sequencing methods to describe the venoms of two related Drosophila endoparasitoids with distinct infection strategies, *Leptopilina boulardi* and *L. heterotoma*. Using RNA-seq, we assembled and quantified libraries of transcript sequences from female wasp abdomens. Next, we used mass spectrometry to sequence peptides derived from dissected venom gland lumens. We then mapped the peptide spectral data against the abdomen transcriptomes to identify a set of putative venom genes for each wasp species. Our approach captured the three venom genes previously characterized in *L. boulardi* by traditional cDNA cloning methods as well as numerous new venom genes that were subsequently validated by a combination of RT-PCR, blast comparisons, and secretion signal sequence search. Overall, 129 proteins were found to comprise *L. boulardi* venom and 176 proteins were found to comprise *L. heterotoma* venom. We found significant overlap in *L. boulardi* and *L. heterotoma* venom composition but also distinct differences that may underlie their unique infection strategies. Our joint transcriptomic-proteomic approach for endoparasitoid wasp venoms is generally applicable to identification of functional protein subsets from any non-genome sequenced organism.

## Introduction

The fruit fly *Drosophila melanogaster* is a genetic model system that has been important in the study of conserved aspects of host innate immune responses against pathogens [1]. This work has proven useful for understanding homologous aspects of human innate immunity, such as the role of Toll-like receptors in mounting humoral immune responses against microbes [2], and has been particularly useful for understanding homologous immune defenses in important insects such as vectors of human disease, crop pollinators, and agricultural pests [3]–[5]. However, many aspects of innate immunity in this model system remain incompletely characterized, especially with respect to cellular immune responses [6]–[8].

Some of the most common pathogens of fruit flies are endoparasitoid wasps, which can infect greater than 50% of flies in natural populations [9]–[11]. These wasps lay their eggs in fruit fly larvae and pupae, and the developing wasp larvae eventually consume and kill their fly hosts. However, wasp infection of *D. melanogaster* larvae induces a cellular immune response against wasp eggs termed melanotic encapsulation [6], [12]. Encapsulation of foreign tissues is an important aspect of parasite resistance for a wide diversity of arthropods, including insect vectors of human disease [13], [14]. Furthermore, endoparasitoid wasps produce cocktails of virulence proteins in their venom glands that they inject into the fly body cavity along with their eggs to suppress this fly immune response, leading to an evolutionary arms race between fly immunity and wasp virulence. Much can be learned about host immune systems by studying the mechanisms that pathogens use to suppress them. Given the genetic tools available in *D. melanogaster*, the identification of venoms from Drosophila endoparasitoids would allow for detailed studies on the molecular biology and evolution of interacting host immune proteins and parasite virulence proteins.

Our goal in this study was to characterize the venom of two well-studied Figitid endoparasitoid wasps, *L. boulardi* and *L. heterotoma*. *L. boulardi* is a specialist on Drosophila species of the melanogaster subgroup while *L. heterotoma* is more of a generalist of the Drosophila genus [15]. Furthermore, these wasps have widely differing infection strategies: *L. boulardi* relies on both cloaking its eggs from host hemocytes and preventing hemocytes from binding to egg surfaces; *D. melanogaster* hosts infected by *L. boulardi* mount strong transcriptional immune responses even during a successful wasp infection. On the other hand, *L. heterotoma* infection relies on host hemocyte destruction; *D. melanogaster* hosts infected by *L. heterotoma* fail to mount any obvious immune transcriptional response at all [15]–[24].

One approach that has been used to identify venom proteins from Drosophila endoparasitoids is the cloning of cDNAs derived from venom gland mRNAs. This traditional cloning approach has resulted in the identification of four venom genes from Drosophila endoparasitoid wasps: *LbGAP* (a RhoGAP domain containing protein), a serpin, and an extracellular superoxide dismutase from the Figitid *L. boulardi*, and an aspartylglucosaminidase from the Braconid *Asobara tabida* [20], [25]–[27]. LbGAP has been shown to alter fly host hemocyte morphology and function, the serpin and superoxide dismutase have been shown to suppress host production of melanin, and the aspartylglucosaminidase has no known function. However, the number of proteins in the venom cocktails of other parasitoid wasps and in a wide range of other venomous animals is on the order of tens or even hundreds [28]–[31], suggesting we have only scratched the surface of Drosophila endoparasitoid wasp venom gene identification.

In order to devise an efficient strategy for identification of total venom protein content in Drosophila endoparasitoid wasps, we first considered the strategies used in other wasp venom studies. Venom proteins from the endoparasitoid wasp *Pteromalus puparum* were identified by mass spectrometry [32], but due to the absence of *P. puparum* transcript sequence data only 12 of the 56 putative venom proteins could be positively identified, based on homology to known proteins. To get around this problem, partial transcript sequences (ESTs) were used in conjunction with proteomics data to investigate the venom of the endoparasitoid wasp *Chelonus inanitus* [33]. This approach identified 29 venom genes, including several genes unique to *C. inanitus* as well as several genes that have no known homology. Similarly, next generation sequencing of venom gland RNAs combined with a low throughput proteomics approach led to the identification of 8 full-length venom transcripts from the wasp *Microctonus hyperodae* [34]. Finally, results from mass spectrometry of *Nasonia vitripennis* venom proteins were mapped back to the completed *N. vitripennis* genome leading to identification of 79 venom genes [35]. Altogether, these studies show that a joint nucleic acid and amino acid sequencing approach is invaluable, and that high throughput sequencing techniques result in a greater ability to identify venom genes.

Here, we took a new high-throughput approach to identify venom proteins from two non-genome sequenced Drosophila endoparasitoid wasps. In our approach, we collected pure venom from wasp venom gland lumens and used liquid chromatography-tandem mass spectrometry to sequence thousands of peptides from the venom protein pool. In order to identify the full-length protein sequences using these peptide sequences, we used next-generation sequencing technology and bioinformatics tools to assemble a transcript library against which peptide sequences could be matched. Venom peptide sequences that map to particular wasp transcript sequences mark those transcripts as venom gene transcripts. This strategy is an efficient means of identifying wasp venoms because all peptide sequences should be real venom peptides, and because modern peptide and nucleic acid sequencing technologies produce phenomenal quantities of data in one pass. Our method should be applicable to any project focused on identification of secreted proteins from non-genome sequenced organisms.

## Results

### Integrating Transcriptome and Proteome Data to Find a Putative Set of Venom Proteins

**Figure 1:**
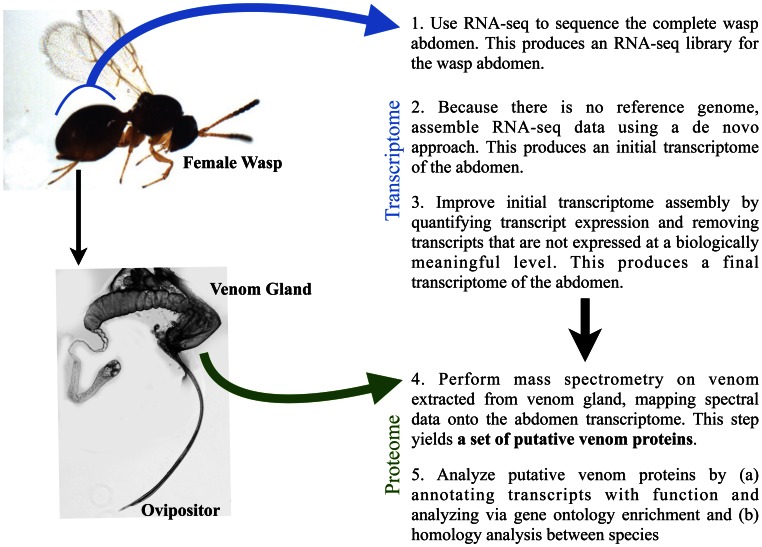
Our method for identifying endoparasitoid wasp venoms.

We pursued a joint transcriptomic and proteomic strategy for analyzing *L. boulardi* and *L. heterotoma* venom content (Fig. 1). We first performed RNA-seq on female abdomens of each wasp species. The abdomen contains the venom gland and at approximately 1 mm long is the smallest tissue containing the venom gland that can be easily collected in mass quantities. However, abdomen tissue samples will still contain transcripts from gut, fat body, body muscle, and reproductive tract tissues in addition to all the transcripts from the venom gland itself. Total RNA was isolated from abdomen samples, poly(A) mRNAs were pulled down with the use of oligo(dT) beads, and mRNAs were processed into cDNAs for sequencing. We obtained two sets of sequences from each sample, first using an Illumina GA-II to produce 50 bp single-end reads and then using an Illumina Hi-Seq 2000 to produce 2×100 bp paired-end reads. This sequencing produced 16.5 Gb of data for *L. boulardi* and 21.9 Gb for *L. heterotoma*.

Because there is no reference genome for *L. boulardi* or *L. heterotoma*, we performed de novo assembly of transcripts using Trinity version r2011-10-29 [36] to create an abdomen transcriptome from females of each wasp species. Trinity assembles contigs via overlaps amongst reads. Initial assemblies for both wasps produced a very large number of potential transcripts, almost 50,000 for *L. boulardi* and more than 270,000 for *L. heterotoma* (Table 1). Assembly statistics show relatively large N50 values of 2142 bp for *L. boulardi* and 1653 bp for *L. heterotoma* (meaning that half of the total assembled sequence is found in transcripts of at least that length) but small mean lengths, indicating that the assemblies include a large number of short transcripts. Mapping the RNA-seq reads back to the assemblies revealed that the assemblies incorporated most of the read data. For *L. boulardi*, 96% of single-end reads and 88% of paired-end reads mapped back to the assembly; for *L. heterotoma*, 95% of single-end reads and 86% of paired-end reads mapped back to the assembly.

**Table 1:** Assembly statistics for the abdomen transcriptomes of each species.

		# Transcripts >100 bp	N50 (bp)	Mean Length (bp)
***L. boulardi***	Initial Assembly	49,972	2142	390
	Expression-filtered Assembly	21625 (43% of initial set)	3856	1411
***L. heterotoma***	Initial Assembly	269,692	1653	339
	Expression-filtered Assembly	32293 (12% of initial set)	5674	1524

To filter out assembly artifacts and incomplete/short transcripts, we calculated expression levels for each contig using RSEM [37] and removed transcripts that were not sufficiently expressed (Table 1). RSEM probabilistically maps reads to transcripts, calculates expression based on the mapped reads, and reports expression in units of transcripts per million (TPM). Only paired-end reads were used with RSEM because they can be mapped more accurately and hence yield more accurate expression levels. Using the recommended threshold of 1 TPM, a set of expressed transcripts was identified: 43% of *L. boulardi* transcripts and only 12% of *L. heterotoma* transcripts were sufficiently expressed, indicating that many of the original contigs were of low quality. Filtering the assemblies to include only expressed transcripts markedly improved the assemblies, as N50 and mean length both improved dramatically, especially for *L. heterotoma* (Table 1). Thus, expression filtering provided a concise library of putative transcripts from female wasp abdomens. We then translated each transcript sequence from the abdomen transcript libraries of each wasp species in all possible frames, to be used as database against which our proteomics data could be compared.

We used liquid chromatography-tandem mass spectrometry to identify peptide sequences from pools of venom purified from the lumens of wasp venom glands. This procedure resulted in 2,917 *L. boulardi* peptide sequences and 3,625 *L. heterotoma* peptide sequences (Table 2). The program SEQUEST was then used to query these peptide sequences against the wasp abdomen translated transcript databases. Approximately 90% of all venom peptides from each wasp species had unique hits to the abdomen transcript databases and also hit transcripts that were hit by at least one other peptide (Table 2). We considered transcript sequences that were hit by more than one of the filtered venom peptides to code for genuine venom proteins, and this analysis yielded 129 putative venom proteins for *L. boulardi* and 176 venom proteins for *L. heterotoma* (Datasets S1–S4). These putative venom proteins were hit by an average of approximately 20 peptide sequences and average peptide coverage across the full protein sequences was approximately 23% for both wasp species (Table 2). The fact that thousands of peptide sequences queried against tens of thousands of transcript sequences repeatedly hit only 129 and 176 *L. boulardi* and *L. heterotoma* transcript sequences, respectively, indicates that the proteins collected from wasp venom glands represent a unique and specific subset of the total set of proteins made in female wasp abdomens, as expected.

**Table 2:** Proteomics results.

	# Peptides	# SEQUEST-filtered Peptides	# Venom Proteins	Avg. # Peptide Hits Per Venom Protein	Venom Protein Coverage (%)
***L. boulardi***	2917	2686 (92% of initial set)	129	21.0	23.7
***L. heterotoma***	3625	3252 (90% of initial set)	176	18.5	23.2

### Expression Specificity of Putative Venom Transcripts

As one means of validating the putative venom proteins, we assayed the tissue specificity of their transcription using RT-PCR. We reasoned that most venom genes would be expressed only in female wasps and specifically in venom glands, although some previously identified Hymenopteran venoms, such as Calreticulin and Heat-shock protein 70 [38], [39] are homologous to essential genes from other organisms and therefore may be globally expressed.

**Figure 2:**
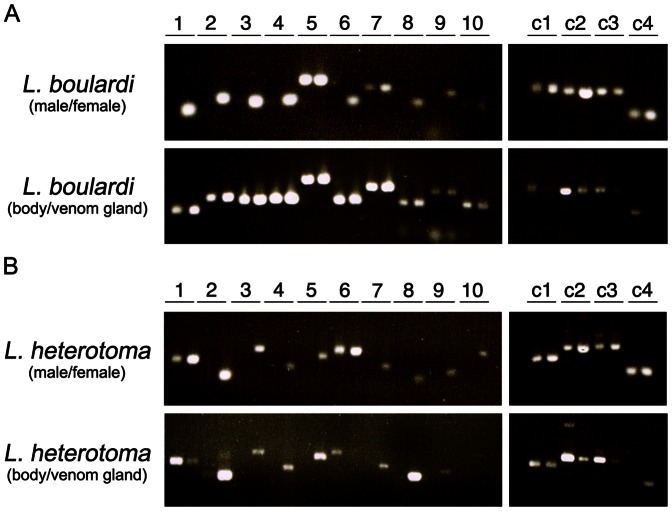
RT-PCR of putative venom genes from *L.* *boulardi* (A) and *L. heterotoma* (B). Sex- (top panel) and tissue- (bottom panel) specific RT-PCRs of venom (1–10) and control (c1–c4) genes. Gene ID numbers correspond to those listed in Tables 3 and 4.

We randomly selected ten putative venom genes from each of *L. boulardi* and *L. heterotoma*, along with a panel of four non-venom control genes (Tables 3 and 4), and attempted to amplify them from adult male and female wasp cDNAs (Fig. 2, top panel in A and B). In both cases, 8 out of 10 venom genes were specifically expressed in females, suggesting that they are linked to parasitism. In both wasps, the functions of the non-female specific genes (arginine kinase and glucose dehydrogenase in *L. boulardi*, and arginine kinase and insulin-like growth factor binding protein in *L. heterotoma*), as well as a lack of other homologous sequences in the respective transcriptomes, suggests they may be essential, and therefore unlikely to be venom specific, although this does not exclude them from having a virulence function.

**Table 3:** Venom and control genes for RT-PCR from *L. boulardi*.

ID	Sequence ID	Annotation	Peptide hits
*Venom genes*			
*1*	*serpin*	*Serpin*	*15*
2	comp225_c0_seq1	Complement binding protein	8
3	comp233_c0_seq1	RhoGAP	18
*4*	*LbGAP*	*RhoGAP*	*13*
5	comp500_c0_seq1	Arginine kinase	18
6	comp1645_c0_seq15	Lipase A	17
7	comp2409_c0_seq1	Glucose dehydrogenase	29
8	comp4291_c0_seq2	–	40
9	comp4434_c0_seq1	Glycosyl hydrolase	17
10	comp9004_c0_seq1	Pentapeptide repeat protein	18
*Control genes*			
*c1*	*elav*	pan-neuronal	*–*
*c2*	*His2a*	ubiquitous	*–*
*c3*	*colIV*	Ubiquitous	*–*
*c4*	*RNApolII*	Ubiquitous	*–*

**Table 4:** Venom and control genes for RT-PCR from *L. heterotoma*.

ID	Sequence ID	Annotation	Peptide hits
*Venom genes*			
1	comp111_c0_seq1	Insulin-like growth factor-binding protein	9
2	comp155_c0_seq1	RhoGAP	17
3	comp1495_c0_seq1	–	19
4	comp1323_c0_seq2	Neprilysin	14
5	comp1525_c0_seq1	LOC100119416	12
6	comp1824_c0_seq1	Arginine kinase	12
7	comp2240_c0_seq1	–	25
8	comp3755_c0_seq1	Inosine-uridine preferring nucleoside hydrolase	11
9	comp3668_c0_seq1	–	6
10	comp4159_c1_seq1	–	21
*Control genes*			
*c1*	*elav*	pan-neuronal	*–*
*c2*	*His2a*	ubiquitous	*–*
*c3*	*colIV*	ubiquitous	*–*
*c4*	*RNApolII*	ubiquitous	*–*

We next amplified the panel of genes from cDNAs made from dissected venom glands and non-venom ‘body’ samples (female caracasses following venom gland dissection) (Fig. 2, bottom panel in A and B). Surprisingly, all of the *L. boulardi* venom genes were expressed in both venom gland and body samples, including the two previously identified venom genes LbGAP and serpin. This suggests that these *L. boulardi* venom genes may serve distinct roles in other tissues but behave as virulence factors when expressed in the venom gland. It is also possible that *L. boulardi* virulence genes may be transcribed outside of the venom gland, for example in the ovaries, similar to another Drosophila parasitoid, *Asobara japonica* [40].

From the *L. heterotoma* samples, all 8 female specific genes were found to be specifically expressed in the venom gland (note there is a weak venom gland band in sample 10). Of the two *L. heterotoma* venom genes expressed in both males and females, one was expressed in both female body samples and in the venom gland (insulin-like growth factor binding protein, sample 1), while the second (arginine kinase, sample 6) was found in the female body sample only. However arginine kinases are found in the venoms of several wasp species including *L. boulardi*, *P. puparum* and *Cyphononyx dorsalis* [32], [41] and represented one of the more abundant proteins in our mass spectrometry data (Table 4), so it remains possible that the arginine kinase is a venom protein despite its apparent absence from our venom gland RT-PCR assay. Finally, all control wasp genes showed expression in both males and females, and most showed expression in venom glands as well. Overall, this expression data is consistent with the idea that most of our predicted venom genes show specific expression, either in venom glands (*L. heterotoma*) or in female wasps (*L. boulardi*).

### Homology to Known Venoms

**Figure 3:**
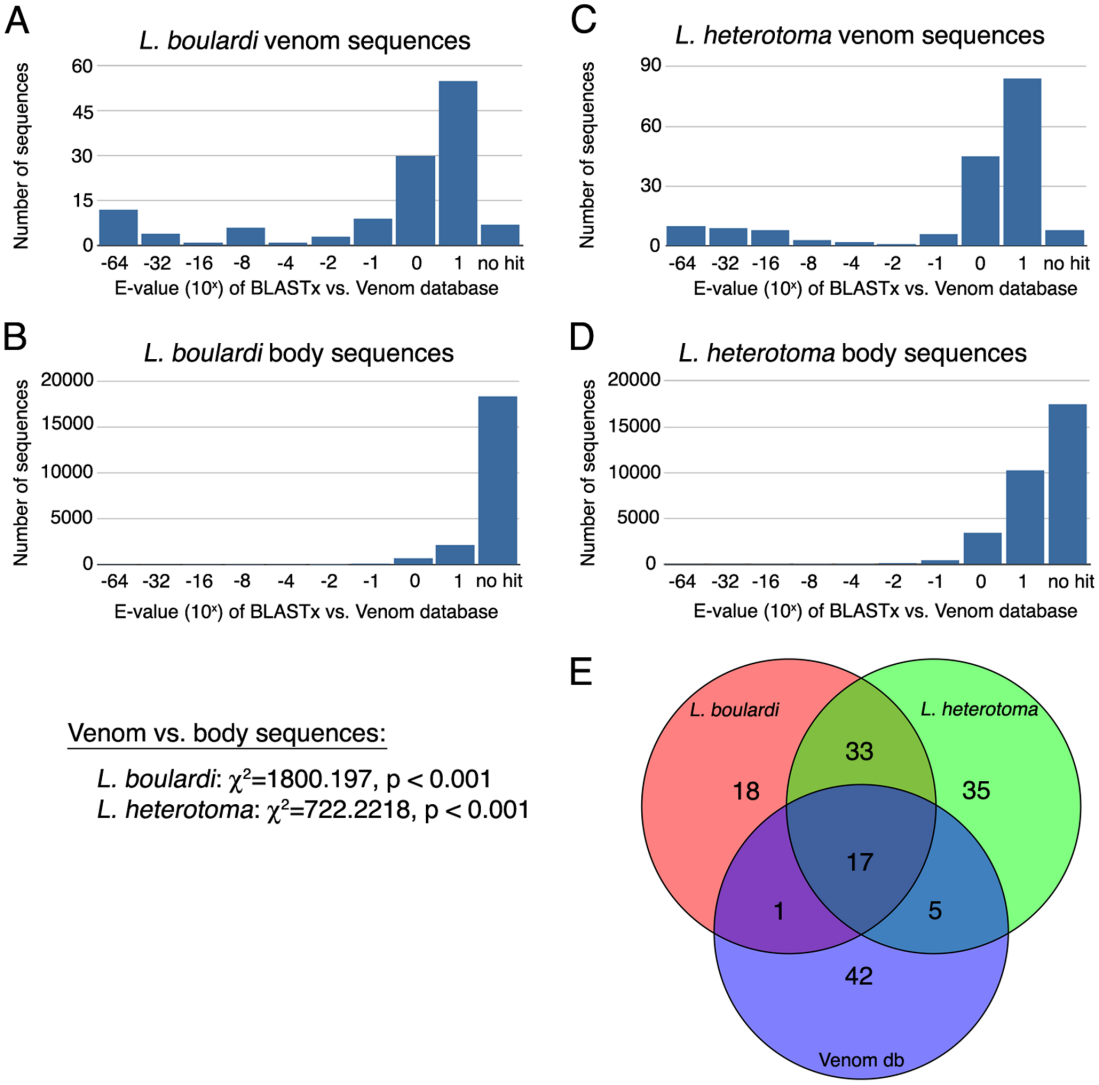
E-value distributions of BLASTx results of indicated transcript subset blasted against the known venom database: (A) *L.* *boulardi* venom, (B) *L. boulardi* body, (C) *L. heterotoma* venom, and (D) *L. heterotoma* body sequences. Venom and body E-value distributions were compared using Chi-squared tests. (E) Venn diagram illustrating overlap of unique conserved domains identified from *L. boulardi* and *L. heterotoma* venoms and the known venom database.

Another means of validating the putative venom proteins that we identified is to examine homology between our venom proteins and known Hymenopteran venom proteins. Based on the existing Hymenopteran venom literature, we would expect a significant degree of overlap among venom proteins of different species. To test this, we blasted wasp transcript libraries corresponding both to the identified venom proteins and the non-venom ‘body’ proteins using BLASTx to a database of known Hymenopteran venom proteins extracted from GenBank (868 proteins from 89 different species, database available upon request) and plotted the E-value distributions. We found that venom transcripts from *L. boulardi* and *L. heterotoma* have significantly lower (more significant) BLASTx E-values when queried against the venom database than their respective body transcripts (Fig. 3A–D). This analysis shows that *L. boulardi* and *L. heterotoma* venoms have homology to other Hymenopteran venoms, and provides further evidence that our approach has led to the successful identification of venom proteins.

To further characterize the overlap between venoms, we used the conserved domain database (CDD) [42] to identify putative functional domains in the venom proteins of *L. boulardi*, *L. heterotoma*, and the Hymenopteran venom database. We identified 69 unique conserved domains in the 129 *L. boulardi* venom proteins, 90 unique conserved domains in the 176 *L. heterotoma* venom proteins, and 65 unique conserved domains in the 868 venom proteins that make up the Hymenopteran venom database. There is a great deal of overlap among these conserved domain predictions (Fig. 3E), both among all three lists, and between *L. boulardi* and *L. heterotoma* specifically. Interestingly, there are far fewer domains found in the venom database that are shared with only one of *L. boulardi* or *L. heterotoma*. This suggests that there is a conserved subset of venom proteins (and likely venom activities) shared across Hymenopteran species, but that many venom activities are also unique to species.

### Analysis of Putative Venom Functions

Wasp venom gland cells secrete venom into the lumen of the gland in preparation for laying eggs in a host, and hence we expected that putative venom proteins would often include secretory signals in their amino acid sequence. We used the SignalP program [43] to find secretory signals in the open reading frames of venom and body proteins (Table 5). For both wasp species, putative venom proteins included secretory signals much more frequently than did body proteins. In *L. boulardi*, 30% of venom proteins included secretory signals while only 5% of body proteins included secretory signals (chi square P<10^−4^). Similarly, 45% of *L. heterotoma* venom proteins and 6% of body proteins included secretory signals (chi square P<10^−4^). The significantly heightened presence of secretory signals in our lists of putative venom proteins is further validation that our venom identification strategy was successful.

**Table 5:** Analyzing venom and body open reading frames for secretion signal sequences, PFAM and gene ontology (GO) annotations, and molecular function enrichment analyses based on GO annotations.

	Secretory Signal	PFAM	PFAM to GO	Enrichment Resultsp_bonferroni_ <0.05
***L. boulardi,*** **venom**	39/129 (30%)	87/129(67%)	68/87(78%)	• oxidation-reduction process (GO:0055114)• lipid transporter activity (GO:0005319)• glycolysis (GO:0006096)• oxidoreductase activity, acting on CH-OH group of donors (GO:0016614)• antioxidant activity (GO:0016209)
***L. boulardi*** **, all**	1172/21625 (5%)	9033/21625 (42%)	6949/9033 (77%)	
***L. heterotoma*** **, venom**	80/176 (45%)	133/176 (76%)	102/133 (77%)	• guanyl ribonucleotide binding (GO:0032561)• GTP binding (GO:0005525)• guanyl nucleotide binding (GO:0019001)• GTPase activity (GO:0003924)• hydrolase activity (GO:0016787)• acid phosphatase activity (GO:0003993)• glycolysis (GO:0006096)• hydrolase activity, acting on acid anhydrides (GO:0016817)• nucleoside-triphosphatase activity (GO:0017111)• nucleic acid binding (GO:0003676)• pyrophosphatase activity (GO:0016462)• hydrolase activity, acting on acid anhydrides, in phosphorus-containing anhydrides (GO:0016818)
***L. heterotoma*** **, all**	1840/32293 (6%)	11488/32293 (36%)	8766/11372 (77%)	

To better understand functions of the putative venom proteins, we performed an enrichment analysis using gene ontology (GO) annotations (Tables 5 and S1). Whereas a majority of venom transcripts could be assigned to conserved protein families (avg. 72%) this was only possible for a minority of the total transcripts (avg. 39%). This difference was expected given that venom transcripts have all been verified using protein spectral data. For genes assigned to protein families, similar proportions of venom transcripts and total transcripts could be assigned a GO annotation (Table 5).

The known venom proteins were not included within the enriched GO terms in *L. boulardi* venom. This suggests that rather than clustering into specific functions, virulence genes may encode a broad range of activities, and thus target multiple host mechanisms. Interestingly we found that *L. boulardi* venom is enriched in proteins classified as having ‘antioxidant activity’ (GO:0016209) (Table 6). *D. melanogaster* larvae are thought to generate oxygen radicals as part of the immune response [44]–[47], and perhaps the antioxidant proteins in *L. boulardi* venom counteract this defense.

**Table 6:** *L. boulardi* venom proteins with annotated ‘antioxidant’ activity (GO:0016209).

*Annotation*	*Sequence ID*
Peroxiredoxin-1	comp193_c0_seq1
Protein disulfide-isomerase	comp692_c0_seq1
Peroxiredoxin-6	comp3099_c0_seq1

Other parasitoid wasps have been shown to alter host physiology and metabolism [48], [49]. We found that the only enriched GO term shared between *L. boulardi* and *L. heterotoma* venom is ‘glycolysis’ (GO:0006096)(Table 7), and that additionally *L. boulardi* venom is also enriched in ‘lipid transporter activity’ (GO:0005319). It seems unlikely that glycolytic enzymes or lipid transport molecules would play a role in parasitoid virulence, but their presence supports the idea that *L. boulardi* and *L. heterotoma* venoms may similarly regulate host metabolism.

**Table 7:** Wasp venom proteins with annotated ‘glycolysis’ activity (GO:0006096).

*Wasp species*	*Annotation*	*Sequence ID*
*L. boulardi*	Fructose-bisphosphate aldolase	comp413_c1_seq2
*L. boulardi*	Pyruvate kinase	comp431_c0_seq1
*L. boulardi*	Enolase	comp464_c0_seq1
*L. boulardi*	Phosphoglycerate kinase	comp1646_c0_seq1
*L. heterotoma*	Fructose-bisphosphate aldolase	comp307_c0_seq1
*L. heterotoma*	Pyruvate kinase	comp576_c0_seq1
*L. heterotoma*	Enolase	comp644_c0_seq1
*L. heterotoma*	Phosphoglycerate kinase	comp2171_c0_seq1

We found four related enriched GO terms in *L. heterotoma* venom, ‘guanyl ribonucleotide binding’ (GO:0032561), ‘GTP binding’ (GO:0005525), ‘guanyl nucleotide binding’ (GO:0019001) and ‘GTPase activity’ (GO:0003924). The GTPase activity category is composed of 12 genes, including homologs of the elongation factors EF1 and EF2, and a family of 10 related genes that are homologous to a single gene in the genome-sequenced wasp *Nasonia vitripennis* (LOC100119416). The expansion of this protein family in *L. heterotoma* appears to be evolutionarily recent as the *L. boulardi* transcriptome does not contain any homologous genes, and members of this family have not been found in any other Hymenopteran venoms to date. Interestingly, host Rho GTPases play major roles in blood cell migration, adhesion, and spreading, and the *D. melanogaster* small Rho GTPases Rac1 and Rac2 are required for the successful encapsulation of wasp eggs [50]–[54]. Although the exact roles of *L. heterotoma* venom GTPases in the fly-wasp interaction cannot be known without experimentation, their identification shows the utility of our high throughput, unbiased venom identification approach, and this result provides a specific basis for further mechanistic study.

### Comparison of Species Venom Transcripts

**Figure 4:**
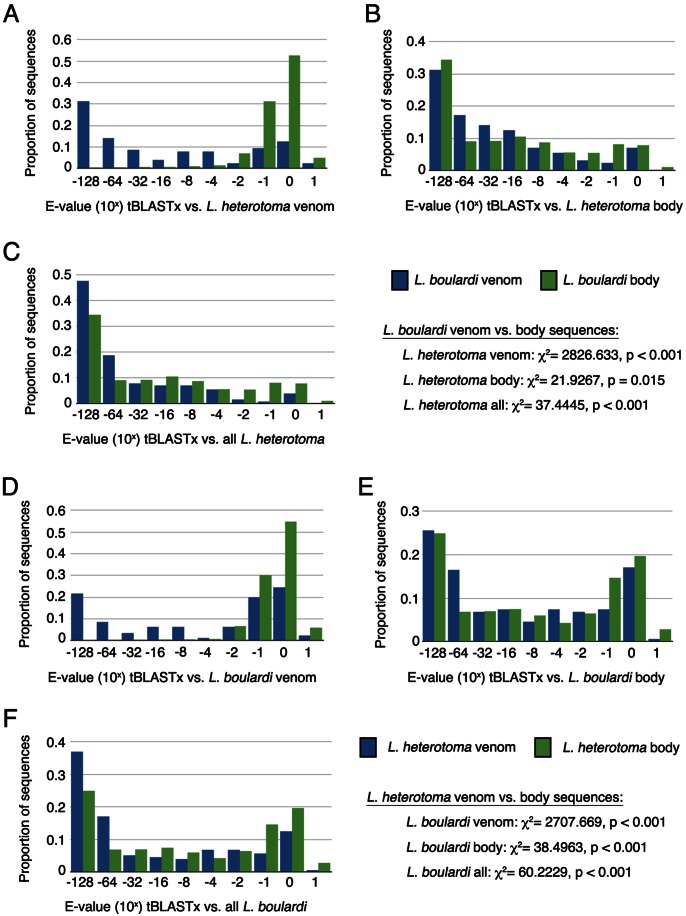
E-value distributions of tBLASTx results of the indicated transcript subset comparisons. (A–C) *L. boulardi* venom (blue) and body (green) transcripts blasted against *L. heterotoma* venom (A), body (B) and all (C) sequences. (D–F) *L. heterotoma* venom (blue) and body (green) blasted against *L. boulardi* venom (D), body (E) and all (F) sequences. E-value distributions were compared by Chi-squared tests.

Our transcriptome data allows us to investigate the amount of sequence homology between *L. boulardi* and *L. heterotoma*, and particularly the homology between venom genes. We aligned the venom and body transcriptome subsets of each wasp to the venom, body and complete transcriptomes of the other using tBLASTx and compared the distributions of E-values. We found that *L. boulardi* and *L. heterotoma* venom sequences are significantly more similar to each other (lower tBLASTx E-values, blue bars in Fig. 4A and 4D) than the body sequences are to venom sequences (green bars in Fig. 4A and 4D), and found large tails (>20%) of venom sequence comparisons in the highest homology (lowest E-score) category. These data confirm that our venom gland lumen proteomic approach led to the identification of similar, functionally relevant subsets of venom genes from each species, and that there is a high degree of sequence homology among Leptopilina venoms.

There are also significant differences in E-value distributions when comparing the venom versus body transcript subsets of one wasp to body sequences of the other (Fig. 4B and 4E), and when comparing venom versus body transcript subsets of one wasp to the full transcriptome of the other (Fig. 4C and 4F). Venom sequences show higher homology than body sequences to whatever transcript set they are blasted against. These data suggest that body transcripts, which have not been validated by being matched to wasp protein sequences, may include a significant fraction of erroneous contigs. Consistent with this hypothesis, our transcript assembly and expression validation pipeline yielded one third more *L. heterotoma* transcripts than *L. boulardi* transcripts (Table 1), suggesting the *L. heterotoma* library may contain more false contigs, and blast analyses using *L. heterotoma* as the query species (Fig. 4E, 4E, and 4F) tended to show greater tails of sequence comparisons in the lowest homology (greatest E-score) categories. However, the existence of erroneous contigs in the body transcript subsets cannot alone explain the high homology observed between *L. boulardi* and *L. heterotoma* venom transcripts, as the differences in venom versus body E-value distributions are much greater in venom comparisons (Fig. 4A and 4D) than in other comparisons (Fig. 4B, 4C, 4E, and 4F).

### Identification of Known Leptopilina Venom Proteins

**Figure 5:**
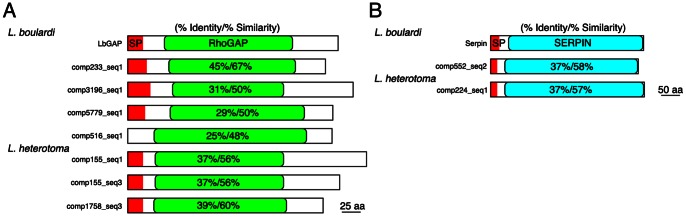
Novel homologs of known *L.* *boulardi* venom proteins in *L. boulardi* and *L. heterotoma*. (A) % identity and % similarity of novel homologs of the venom LbGAP in *L. boulardi* and *L. heterotoma* venoms within the RhoGAP domain (green box) as indicated. (B) % identity and % similarity of novel homologs of the venom Serpin in *L. boulardi* and *L. heterotoma* venoms within the serpin domain (blue box) as indicated. Signal peptides are shown in red.

To further evaluate the predicted venom proteins, we used BLASTp to compare our predicted venom proteins with previously known Leptopilina venom proteins. Our approach successfully identified all three known *L. boulardi* venom proteins (Table 8): LbGAP (comp433_c0_seq1), Serpin (comp138_c0_seq1), and Extracellular superoxide dismutase 3 (comp937_c0_seq1) [20], [25], [26]. Furthermore, we found that *L. boulardi* has four additional RhoGAP domain containing proteins in its venom that show strong homology to LbGAP, and a second previously unidentified venom Serpin protein (Fig. 5). This suggests that RhoGAP and Serpin activities play an important role in *L. boulardi* virulence.

**Table 8:** *L. boulardi* venom proteins showing homology to known venom proteins.

Annotation	Sequence ID	Best blast hit (species)	E-value
*Known L. boulardi venom proteins*			
Extracellular superoxide dismutase 3	comp937_c0_seq1	*L. boulardi*	*9.00E-99*
RhoGAP	comp433_c0_seq1	*L. boulardi*	*2.00E-126*
Serpin	comp138_c0_seq1	*L. boulardi*	*3.00E-155*
*Other venom proteins*			
RhoGAP	comp233_c0_seq1, comp516_c0_seq1, comp3196_c0_seq1, comp5779_c0_seq1	*L. boulardi*	*5.00E-46*
Serpin	comp552_c0_seq2	*L. boulardi*	*8.00E-66*
Arginine kinase	comp500_c0_seq1	*Cyphononyx dorsalis*	*0.0*
Calreticulin	comp263_c0_seq1	*Nasonia vitripennis*	*2.00E-157*
Cysteine-richvenom protein 6	comp242_c2_seq28	*Pimpla hypochondriaca*	*9.00E-09*
Glucose dehydrogenase venom protein	comp2100_c0_seq1, comp2409_c0_seq1	*Nasonia vitripennis*	*4.00E-98*
Inosine-uridine preferring nucleoside hydrolase	comp3592_c0_seq1	*Nasonia vitripennis*	*4.00E-57*
Lipase A	comp1645_c0_seq14, comp1645_c0_seq15	*Nasonia vitripennis*	*1.00E-35*
Tyrosine 3/tryptophan5-monooxygenase	comp31_c0_seq1	*Eumenes pomiformis*	*1.00E-71*
Venom metalloprotease	comp3680_c0_seq1	*Eulophus pennicornis*	*4.00E-11*
Venom acid phosphatase	comp9544_c0_seq1	*Bombus impatiens*	*1.00E-69*
Venom chitinase	comp435_c0_seq1	*Chelonus inanitus*	*1.00E-111*
Venom protein 1	comp6910_c0_seq1	*Microctonus hyperodae*	*6.00E-15*
Venom protein 8	comp953_c0_seq1	*Microctonus hyperodae*	*3.00E-14*

There were no previously characterized *L. heterotoma* venom proteins, but interestingly, we found that *L. heterotoma* venom contains homologs of two of the known *L. boulardi* venom proteins: three LbGAP-like proteins and one Serpin-like protein (Table 9, Fig. 5). Besides LbGAP and Serpin, *L. boulardi* and *L. heterotoma* share many other venom protein molecular functions (Tables 8 and 9). For example, both venoms contain an Arginine kinase, a protein type demonstrated to have paralytic activity in the parasitic spider wasp [41], and Calreticulin, which has been shown to inhibit hemocyte spreading and encapsulation in a butterlfy wasp endoparasitoid [39]. However, we found that *L. heterotoma* has approximately 25% more venom genes than does *L. boulardi* (Table 2), twelve of which are GTPases (Tables 5 and S1), and these extra venom proteins may enable *L. heterotoma* to infect a greater diversity of host species than *L. boulardi* [15] and may also be responsible for their differences in infection characteristics in their common natural host, *D. melanogaster* [15]–[24]. Altogether, our data suggest that there are a common set of venom protein functionalities that most parasitic wasps tend to use, but that even closely related species such as *L. boulardi* and *L. heterotoma* can evolve unique venom activities suited to their particular niches.

**Table 9:** *L. heterotoma* venom proteins showing homology to known venom proteins.

Annotation	Sequence ID	Best blast hit (species)	E-value
Arginine kinase	comp1824_c0_seq1	*Cyphononyx dorsalis*	*0.0*
Aspartylglucosaminidase	comp18_c0_seq1, comp6288_c0_seq1	*Nasonia vitripennis*	*3.00E-93*
Calreticulin	comp326_c0_seq1	*Nasonia vitripennis*	*7.00E-153*
Glucose dehydrogenase venom protein	comp4489_c2_seq4	*Nasonia vitripennis*	*9.00E-121*
Inosine-uridine preferring nucleoside hydrolase	comp3755_c0_seq1	*Nasonia vitripennis*	*9.00E-64*
Lipase A	comp2257_c0_seq1	*Nasonia vitripennis*	*7.00E-34*
Metalloprotease	comp1346_c0_seq1, comp3196_c0_seq1, comp8757_c0_seq1	*Nasonia vitripennis*	*2.00E-20*
Metalloproteinase	comp3216_c0_seq1	*Eulophus pennicornis*	*9.00E-20*
Tyrosine 3/tryptophan 5-monooxygenase	comp455_c2_seq1	*Eumenes pomiformis*	*3.00E-71*
RhoGAP	comp155_c0_seq1, comp155_c0_seq3, comp1758_c1_seq3	*L. boulardi*	*1.00E-25*
Venom acid phosphatase	comp1442_c0_seq1, comp1442_c0_seq2, comp1602_c1_seq12, comp2614_c0_seq1, comp2636_c0_seq1, comp3064_c0_seq1	*Nasonia vitripennis*	*5.00E-78*
Venom allergen 3	comp1331_c0_seq1	*Nasonia vitripennis*	*3.00E-37*
Venom carboxylesterase-6	comp255_c0_seq1	*Apis florea*	*9.00E-118*
Venom protein 1	comp1040_c0_seq26	*Microctonus hyperodae*	*5.00E-54*
Venom protein 5	comp1564_c0_seq1	*Brachymyrmex patagonicus*	*3.00E-51*
Venom protein 8	comp719_c0_seq1	*Microctonus hyperodae*	*4.00E-14*
Venom protein Ci-95	comp128_c2_seq1	*Chelonus inanitus*	*1.00E-28*
Venom serine protease	comp513_c0_seq1	*Nasonia vitripennis*	*4.00E-117*
Serpin	comp224_c1_seq1	*L. boulardi*	*2.00E-62*

## Discussion

Our approach integrated high-throughput transcriptomic and proteomic data to investigate the venoms of two endoparasitoid wasp species, *L. boulardi* and *L. heterotoma*. We first assembled the transcriptomic data and then filtered the assembly based on expression levels; next, we used the filtered assembly as a library for mapping mass spectrometry data to identify a list of putative venom proteins. The putative venom transcripts we identified included the three previously characterized *L. boulardi* venom proteins, and we further validated our venom gene identification approach using experimental and computational techniques. RT-PCR results showed female- and venom gland-specific transcription of putative venom genes, the putative venom proteins from *L. boulardi* and *L. heterotoma* showed significant homology to each other and to other known Hymenopteran venom proteins, and the putative venom proteins were significantly more likely to contain secretion signal sequences.

Our venom identification approach yielded many more proteins than lower throughput methods previously used for analyzing wasp venoms. So far, traditional cloning methods have only identified 4 venom proteins from Drosophila endoparasitoids [19], [20], [25]–[27], but we identified 304 potential venom proteins in two wasp species. Our RT-PCR results showed that 19 of 20 putative venom genes were expressed in venom glands (often specifically in venom glands or females), suggesting a true positive rate of ∼95%, so by extrapolation we conclude that our approach yielded about 122 venom genes for *L. boulardi* and 167 venom genes for *L. heterotoma*. Our venom identification method is applicable to general protein identification in any tissue sample or cell type, but will likely be most useful for subsets of proteins that are spatially sequestered. Whereas transcriptomics alone is likely sufficient for general protein identification in particular tissues, if subsets of proteins are sought, and the protein subsets can be isolated (such as in an organelle or gland), a joint transcriptomic and proteomics approach is ideal for specifically identifying those protein subsets.

Our analysis of venoms from the *D. melanogaster* wasp endoparasitoids *L. boulardi* and *L. heterotoma* provides a foundation for future functional studies of the infection strategies of these wasps. For example, we have identified 9 novel homologs of known *L. boulardi* venom genes across both wasp species (Fig. 5), we have identified numerous *L. boulardi* and *L. heterotoma* protein types that are commonly found in venoms of other Hymenopterans (Table 8 and 9), and we have identified a potentially novel type of venom genes, an expanded family of GTPases, in *L. heterotoma*. Learning how Drosophila parasites evade and suppress the cellular encapsulation response will further our understanding of innate immunity in this model organism, especially the weak links in innate immunity that parasites tend to exploit. The difference in venom contents and effects between the two closely related wasps *L. boulardi* and *L. heterotoma* also suggests that venom identification in further species, genera, and families of Drosophila endoparasitoids will continue to provide novel material for elucidating patterns of parasite virulence evolution.

## Methods

### Wasp Strains

*L. boulardi* strain Lb17 and *L. heterotoma* strain Lh14 were collected in Winters, California in 2002 and have been previously described [15]. Laboratory cultures of these wasps were maintained on *D. melanogaster* and are available upon request.

### mRNA Isolation and CDNA Preparation

Abdomens of approximately 200 female wasps aged 2–7 days were dissected into Trizol reagent (Invitrogen) and incubated at 4°C for 24 hrs. Total RNA was isolated according to manufacturer’s directions with the exception that RNA was mixed with 5 µg glycogen (Invitrogen) prior to precipitation with 1 ml isopropanol per 1 ml Trizol overnight at −20°C. RNA was resuspended in a 100 mM Tris-HCl, 500 mM LiCl, 10 mM EDTA, 5 mM DTT, and 1% LiDS (pH 7.5) solution. Poly(A) RNAs were purified using the Dynabeads mRNA Direct kit (Invitrogen) according to manufacturer’s directions. This led to isolation of 840 ng of *L. boulardi* mRNA (from 25.2 µg of total RNA, 3.3% of total) and 1.2 µg of *L. heterotoma* mRNA (from 32.3 µg of total RNA, 3.7% of total).

cDNAs were synthesized from the isolated wasp mRNAs using the SuperScript II double-stranded cDNA synthesis kit (Invitrogen) according to manufacturer’s directions with the following exceptions. First-strand synthesis was primed by random hexamers (Invitrogen) and the SuperScript II reverse transcription reaction was incubated at 42°C for 1 hr. Following second-strand synthesis, cDNA samples were incubated with 1 µl of 10 mg/ml RNase A (Novagen) at 37°C for 1 hr. The cDNA samples were then extracted with phenol:chloroform and precipitated with 7.5 M ammonium acetate and ethanol. Pellets were washed three times with 70% ethanol and resuspended in 1X TE buffer.

### Venom Protein Isolation

Venom glands from 50 female wasps were dissected into 1X PBS supplemented with 0.5 mM EDTA and Complete Protease Inhibitor Cocktail (Roche) on ice. Tissues were then homogenzied under nonlysing conditions and incubated on ice for a further 5 min. Cells were pelleted by centrifugation at 12000 g at 4°C and venom containing supernatant was removed and combined with 2X sample buffer (4% SDS, 20% glycerol, 120 mM Tris pH 6.8, 0.002% bromophenol blue, 200 mM DTT) for proteomic analysis.

### Tissue Specific RT-PCR

Primers were designed in Primer3 (SDSC Workbench) to amplify control and putative venom genes. Control primers were made to target wasp homologs of *RNA polymerase II*, *elav*, *Histone2A*, and *collagen IV*, and venom primers were made against 10 putative venom genes identified by mass spectrometry of purified venom. Primers used are listed in Tables S2 and S3.

To make tissue specific cDNAs, venom glands from 25 female wasps were dissected in PBS and the venom glands and venom gland-less carcasses were incubated in Trizol reagent for 24 hrs at 4°C. Total RNA was isolated as described above, and reverse transcribed using the QuantiTect reverse transcription kit (Qiagen). Targeted venom and control genes were then amplified from these tissue specific cDNAs. Amplification conditions for venom primers were 95°C for 3 min, 25 cycles of 95°C for 15 sec, 55°C for 30 sec, 72°C for 1 min before a final incubation at 72°C for 1 min, and were the same for control primers except 35 cycles of amplification were used. Amplification products were analyzed on 1% agarose gels.

### Blast Searches

Blast searches were performed using the command line NCBI-BLAST package (version 2.2.25) [55]. The Hymenopteran venom database was assembled from proteins annotated as ‘venom’ and restricted to Hymenoptera on GenBank (as of 05/06/2012). Venom and body transcript subsets were both aligned to the venom database using the BLASTx algorithm with default parameters except that the ‘max target seqs’ parameter was set to 1 to get only the single best blast hit. *L. boulardi* and *L. heterotoma* transcriptomes and transcript subsets were compared using the tBLASTx algorithm with default parameters except that the ‘max target seqs’ parameter was again set to 1. The distributions the single best-hit E-values of venom and body transcript subsets blasted to a common target database were compared to each other by Chi square tests in R (version 2.15.0).

### 1D PAGE Molecular Weight Fractionation and in-gel Tryptic Digestion

Protein extracts were quantified with a BCA Protein Assay Kit (Thermo Scientific). 10 µg protein aliquots from each wasp species were denatured and reduced using Novex Tris-Glycine SDS sample buffer, and separated by molecular weight on a 4–12% Novex Tris-Glycine gel at a constant 125 V for 45 minutes. Gels were stained overnight with a Colloidal Blue Staining Kit at room temperature with gentle agitation, and destained in MilliQ water for more than 3 hrs at room temperature the following day. Each lane was excised into eight molecular weight fractions containing nearly equal staining densities across each band, and in-gel tryptic digestion was carried out with Trypsin Gold following the manufacturer instructions. Digests were reduced in volume to near dryness in a SpeedVac concentrator (Savant) and brought up to 20 µL using a 5% acetonitrile and 0.1% formic acid solution.

### Nano LC-MS(MS)^2^

Peptide digests (10 µL = ∼300 ng, controls calculated at ∼50% efficiency in digestion and peptide recovery) were injected two separate times onto a Surveyor HPLC plus machine (Thermo Scientific, San Jose CA) using a split flow configuration on the back end of a 100 µm internal diameter × 13 cm pulled tip C-18 column (Jupiter C-18 300 Å, 5 µm, Phenomenex). This system was run in-line with a Thermo LTQ XL ion trap mass spectrometer equipped with a nano-electrospray source (Thermo Scientific, San Jose CA), and all data were collected in CID mode. Buffers used were solvent A (0.1% formic acid in ddH_2_O) and solvent B (0.1% formic acid in acetonitrile). The HPLC gradient setting was as follows; 0–10 min using 2% solvent B (98% solvent A), 10–15 min using 12% solvent B, 15–31 min using 30% solvent B, 31–36 min using 36% solvent B, 36–41 min using 45% solvent B, 41–48 min using 65% solvent B, 48–56 min using 95% solvent B, 56–57 min using 2% solvent B, 57–82 min using 2% solvent B. The LTQ collected data on the top 3 ions using a triple play method from 10 to 57 minutes, with a flow rate of 0.6 µl/min. Before and after the analysis window, the spray voltage was 0.0 kV and the flow rate was 3 µl/min. During data collection, the LTQ XL was configured as follows: spray voltage 2.2 kV, capillary temperature 170°C, 1 microscan with a maximum inject time of 25 ms for all modes. For the triple play setup, the SIM scans were obtained in zoom mode using a ±2.5 Da window, and triggered on a signal threshold of 15,000 counts. The MS/MS scans were obtained in normal mode with a minimum signal threshold of 500 counts based on the SIM scan. The activation settings were charge state 5, isolation width 5.0 m/z, normalized collision energy 30.0, activation Q 0.250, and activation time 50.000 ms. For the dependent scans, charge state screening was enabled, and the dynamic exclusion was enabled with the following settings: repeat count 3, repeat duration 20.0 s, exclusion list size 500, and exclusion duration 90.0 s. XCalibur RAW files outputted from the mass spectrometer were collected in profile mode, centroided and converted to MzXML using ReAdW v. 3.5.1 (Institute for Systems Biology). mgf files were then created using MzXML2Search (included in TPP v. 3.5, Institute for Systems Biology) for all scans with a precursor mass between 400 Da and 20,000 Da.

### Protein Identifications and Quality Control

The libraries of female wasp abdomen transcripts were translated in each of three frames in both orientations for each transcript sequence, for a total of six amino acid sequences per mRNA sequence, using the EMBOSS getorf tool, version 6.5.0. The peptide sequence data were then queried against a database containing these translated wasp abdomen transcripts along with common contaminant sequences such as digestion enzymes and human keratin, using SEQUEST (v.27 rev 12,.dta files). The program was set for “no-enzyme”, a precursor mass window of 0.45 Da, trypsin digestion, static modification C at 57.0293, variable modification M at 15.9949, and a fragment-ion mass tolerance of 0.8 Da. The SEQUEST results files were first combined for each of 8 fractions per lane, then filtered using peptide and protein probability [56], grouped by top scoring protein ID, and finally quantified by normalized spectral counts (label free) using ProteoIQ (NuSep, Athens, GA) [57], [58]. The filter cut-off values were set with peptide length (>4 AAs), peptide probability (>0.5), peptides per protein (≥2 peptides), and protein probability (>0.7), and peptides with +1 charge were also excluded from any further analyses. This protocol resulted in a list of venom protein identifications with ≥99.0% confidence.

### Gene Ontology Analysis of Venom Proteins

For all expressed transcripts, the longest open reading frame was found using the EMBOSS getorf tool, version 6.5.0, annotated with PFAM protein families using HMMer version 3.0, and then PFAM annotations were converted to corresponding GO categories using InterPro2GOmapping [59], version date 08/11/2012. The open source GOA tools (https://github.com/tanghaibao/goatools) were used for the enrichment analysis. Results obtained are shown in Table S1.

### Data Access

All mRNA-seq data is available at the DDBJ Sequence Read Archive (http://trace.ddbj.nig.ac.jp/dra/index_e.shtml) under accession number SRA058494. The assembled wasp transcripts were deposited in GenBank under Transcriptome Shotgun Assembly accession numbers GAJA00000000 and GAJC00000000. All raw mass spectrometry data is available at http://www.taylorlab.org/~jeremy/wasp-venom-proteomic-data/. Code bundle at https://bitbucket.org/bxlab/wasp-venom provides data, methods, and original results for transcriptome assembly and quantification, secretion signal sequenceanalysis, and functional annotation and enrichment.

## Supporting Information

Table S1 **Results from gene ontology analysis.** Dots preceding category ID indicate category level relative to top-level category, GO:0003674, molecular function. p_bonferroni_ <0.05
(DOCX)

Table S2 **PCR primers used to amplify** ***L. boulardi*** **genes.**
(DOCX)

Table S3 **PCR primers used to amplify** ***L. heterotoma*** **genes.**
(DOCX)

Dataset S1 **All identified** ***L. boulardi*** **venom cDNAs in Fasta format.**
(FASTA)

Dataset S2 **All identified** ***L. heterotoma*** **venom cDNAs in Fasta format.**
(FASTA)

Dataset S3 **All identified** ***L. boulardi*** **venom proteins in Fasta format.**
(FASTA)

Dataset S4 **All identified** ***L. heterotoma*** **venom proteins in Fasta format.**
(FASTA)
