# Supplementary material for: Integrative Approach Reveals Composition of Endoparasitoid Wasp Venoms
Source: PLoS One. 2013 May 23;8(5):e64125. doi: 10.1371/journal.pone.0064125 (PMC3662768; doi:10.1371/journal.pone.0064125)
Supplement: Table S1 — Results from gene ontology analysis. Dots preceding category ID indicate category level relative to top-level category, GO:0003674, molecular function. pbonferroni <0.05 (DOCX) [file pone.0064125.s001.docx]

Table S1. Results from gene ontology analysis. Dots preceding category ID indicate category level relative to top-level category, GO:0003674, molecular function. p_bonferroni_  < 0.05

L. boulardi

| ID | Representation | Description | Venom Ratio | Body Ratio | p_uncorrected_ | p_bonferroni_ |
| --- | --- | --- | --- | --- | --- | --- |
| ..GO:0055114 | over- | oxidation-reduction process | 15/68 | 434/7195 | 9.19e-06 | 0.00322 |
| ...GO:0005319 | over- | lipid transporter activity | 4/68 | 16/7195 | 1.22e-05 | 0.00427 |
| ....GO:0006096 | over- | glycolysis | 3/68 | 6/7195 | 1.58e-05 | 0.00554 |
| ...GO:0016614 | over | protein binding | 7/68 | 89/7195 | 1.84e-05 | 0.00643 |
| .GO:0016209 | over | antioxidant activity | 4/68 | 28/7195 | 0.000126 | 0.0441 |

L. heterotoma

| ID | Representation | Description | Venom Ratio | Body Ratio | p_uncorrected_ | p_bonferroni_ |
| --- | --- | --- | --- | --- | --- | --- |
| ......GO:0032561 | over- | guanyl ribonucleotide binding | 19/102 | 265/8766 | 1.49e-10 | 7.2e-08 |
| .......GO:0005525 | over- | GTP binding | 19/102 | 270/8152 | 1.49e-10 | 7.2e-08 |
| .....GO:0019001 | over- | guanyl nucleotide binding | 19/102 | 270/8152 | 2.06e-10 | 9.92e-08 |
| .......GO:0003924 | over | GTPase activity | 14/102 | 204/8766 | 1.12e-08 | 5.38e-06 |
| ..GO:0016787 | over- | hydrolase activity | 39/102 | 1460/8766 | 1.35e-07 | 6.52e-05 |
| ......GO:0003993 | over- | acid phosphatase activity | 6/102 | 33/8766 | 1.84e-06 | 0.000886 |
| ….GO:0006096 | over- | glycolysis | 4/102 | 10/8766 | 3.44e-06 | 0.00166 |
| ...GO:0016817 | over- | hydrolase activity, acting on acid anhydrides | 19/102 | 566/8766 | 2.33e-05 | 0.01111 |
| ......GO:0017111 | over- | nucleoside-triphosphatase activity | 18/102 | 537/8766 | 4.03e-05 | 0.0194 |
| ..GO:0003676 | under- | nucleic acid binding | 5/102 | 1715/8766 | 4.08e-05 | 0.0197 |
| .....GO:0016462 | over- | pyrophosphatase activity | 18/102 | 542/8766 | 4.55e-05 | 0.022 |
| ....GO:0016818 | over- | hydrolase activity, acting on acid anhydrides, in phosphorus-containing anhydrides | 18/102 | 548/8766 | 5.26e-05 | 0.0254 |
