# Supplementary material for: Integrative Approach Reveals Composition of Endoparasitoid Wasp Venoms
Source: PLoS One. 2013 May 23;8(5):e64125. doi: 10.1371/journal.pone.0064125 (PMC3662768; doi:10.1371/journal.pone.0064125)
Supplement: Table S2 — PCR primers used to amplify L. boulardi genes. (DOCX) [file pone.0064125.s002.docx]

Table S2. PCR primers used to amplify L. boulardi genes

| Sequence ID | Forward Primer | Reverse Primer |
| --- | --- | --- |
| serpin | CCTAAATTTCGGGTTGAAGC | TCAGCACCATTCTCGTTGAC |
| comp225_c0_seq1 | TCATGAGTGCAATCAGAAGTTTAC | ATTGAAACAAATCCAACAGGTC |
| comp233_c0_seq1 | CAACCAAATCGCGATACTCTC | TGTGTCCAGAAACTGGATGG |
| LbGAP | TGGAATTGCTCCTGAAGACAG | AGACTTGCTTCATCGCTTGG |
| comp500_c0_seq1 | TGTTTGGTGCAACGAAGAAG | TGCATTTCCTTGACAGCTTG |
| comp1645_c0_seq15 | TTATCATTGCTGGCAAATCG | CAACATGGCTTGTTTGCATC |
| comp2409_c0_seq1 | GCTGCTAAGGGTGTTTCGTC | AAACCATGCTTATGGCATTTG |
| comp4291_c0_seq2 | AGCGGATATGGTGGCTAATG | CAGCACATTGGTTGATACCG |
| comp4434_c0_seq1 | TTTCGCAATGGACAGTTCAG | ACTGGTGCTTCCACAACTGG |
| comp9004_c0_seq1 | TTGCAACCCTGGAATTCTTC | TTTCCACCATTCCTTTCTCG |
| elav | AAGCTTAGGCTATGGATTTG | AATCTTATGAATCCAAC |
| His2a | GATTGGCGTCGTCTGTGGTCG | TTGACTTTCAGGTCTTTGG |
| colIV | GAATTGACGGTCGACCTGG | CCGGTCGTCCCATTAGACC |
| RNApolII | ACGCAGAAACCGATCCTATG | ATTCTTGATCCTCGGCAATG |
