# Supplementary material for: Integrative Approach Reveals Composition of Endoparasitoid Wasp Venoms
Source: PLoS One. 2013 May 23;8(5):e64125. doi: 10.1371/journal.pone.0064125 (PMC3662768; doi:10.1371/journal.pone.0064125)
Supplement: Table S3 — PCR primers used to amplify L. heterotoma genes. (DOCX) [file pone.0064125.s003.docx]

Table S3. PCR primers used to amplify L. heterotoma genes.

| Sequence ID | Forward Primer | Reverse Primer |
| --- | --- | --- |
| comp111_c0_seq1 | CCAACATCAACGTTCACCAC | GAGATTTGGACCGACAATGG |
| comp155_c0_seq1 | GAAGCCAAATCGTCCTCTTC | AGCTGAAGCCGATTTCACAG |
| comp1495_c0_seq1 | AAATTGACGGCAAATTCTGG | GCGGTGGCTGTAGAAATAGC |
| comp1323_c0_seq2 | TCGTCCAACCAACTGTTGTC | TGGCACAATGAGAAACATGC |
| comp1525_c0_seq1 | ACGGAATTGGAGGATTTCAG | AACTGTTGCGGACTTTCAGG |
| comp1824_c0_seq1 | AACCAACTTGGGAACGACAG | TGTGTTTGCTTTGGGAGTTG |
| comp2240_c0_seq1 | ATCATTCGGATTTGGAGCTG | ACAACAGCTTTGGTCGGAAC |
| comp3755_c0_seq1 | TATCAAGCCGGAAAGGAATG | CCAAATTGCAAATGCAACAG |
| comp3668_c0_seq1 | TGGAATCATTTCCTCCAACTC | GCCCTCTTCAAAGGCATATC |
| comp4159_c1_seq1 | TGCAGGGAAATGTGTACTGC | GGAGGGTTGACGAAGAATTG |
| elav | TCTTCGTCTACAATTTGGC | CTCGTCCTACGCAGCTTTGC |
| His2a | GATTGGCGTCGTCTGTGGTCG | TTGACTTTCAGGTCTTTGG |
| colIV | GAATTGACGGTCGACCTGG | CCGGTCGTCCCATTAGACC |
| RNApolII | TGAACGACGCTCGTGATAAG | CGCTTTCCCTCTACGTTCTG |
